# The Enhanced Effects of Swimming and Running Preconditioning in an Experimental Model of Myocardial Ischemia/Reperfusion Injury

**DOI:** 10.3390/medicina59111995

**Published:** 2023-11-13

**Authors:** Milos Glisic, Tamara Nikolic Turnic, Vladimir Zivkovic, Bozidar Pindovic, Natalia Vasilievna Chichkova, Vladimir Petrovich Fisenko, Marina Nikolic, Lazar Stijak, Lemina Elena Yurievna, Mirjana Veselinovic, Milena Jovicic, Katarina Mihajlovic, Sergey Bolevich, Vladimir Jakovljevic

**Affiliations:** 1 Department of Physiology, Faculty of Medical Sciences, University of Kragujevac, Svetozara Markovica 69, 34000 Kragujevac, Serbia; miloskg92@gmail.com (M.G.); vladimirziv@gmail.com (V.Z.); marina.rankovic.95@gmail.com (M.N.); drvladakgbg@yahoo.com (V.J.); 2 Department of Pharmacy, Faculty of Medical Sciences, University of Kragujevac, Svetozara Markovica 69, 34000 Kragujevac, Serbia; pindovic.bozidar@gmail.com (B.P.); katarina.radonjic@medf.kg.ac.rs (K.M.); 3 N.A. Semashko Public Health and Healthcare Department, F.F. Erismann Institute of Public Health, I.M. Sechenov First Moscow State Medical University (Sechenov University), 119435 Moscow, Russia; 4 Center of Excellence for Redox Balance Research in Cardiovascular and Metabolic Disorders, 34000 Kragujevac, Serbia; 5 Department of Pharmacology, 1st Moscow State Medical, University IM Sechenov, Trubetskaya Street 8, Str. 2, 119991 Moscow, Russia; fisenko_v_p@staff.sechenov.ru; 6 Department Faculty Therapy, University IM Sechenov, Trubetskaya Street 8, Str. 2, 119991 Moscow, Russia; chichkova_n_v@staff.sechenov.ru; 7 Institute of Anatomy, School of Medicine University in Belgrade, Dr Subotica 4/II., 11000 Belgrade, Serbia; lazar.stijak@gmail.com; 8 1st Moscow State Medical, University IM Sechenov, Trubetskaya Street 8, Str. 2, 119991 Moscow, Russia; 9 Department of Internal Medicine, Faculty of Medical Sciences, University of Kragujevac, Svetozara Markovica 69, 34000 Kragujevac, Serbia; miraveselinovic.m@gmail.com; 10 Clinic for Rheumatology and Allergology, University Clinical Center, 34000 Kragujevac, Serbia; 11 Department of Communication Skills, Faculty of Medical Sciences, University of Kragujevac, 34000 Kragujevac, Serbia; limena27@gmail.com; 12 Department of Human Pathology, 1st Moscow State Medical, University IM Sechenov, Trubetskaya Street 8, Str. 2, 119991 Moscow, Russia; bolevich2011@yandex.ru

**Keywords:** aerobic preconditioning, anaerobic performance, swimming, running, rat, myocardial ischemia

## Abstract

*Background and Objectives*: This study was conducted to examine the influence of different swimming and running protocols as forms of physiological preconditioning on an isolated rat heart’s ischemia/reperfusion injury. *Materials and Methods*: This study was conducted on 60 male Wistar albino rats (6 weeks old, bw: 200 ± 20 g), divided into: CTRL group—a sedentary control group; sAeT—a group that underwent aerobic swimming conditioning using a swimming protocol for 8 weeks; sAnT—a group that underwent anaerobic swimming conditioning; rAeT—a group that underwent aerobic running conditioning; and rAnT—a group that underwent anaerobic running conditioning. After the preconditioning protocols, ex vivo estimating of myocardial function according to the Langendorff technique was performed. *Results*: The anaerobic running training decreased heart rate and the anaerobic swimming training reduced coronary flow, demonstrating the difference in the physiological heart response of aerobic/anaerobic physical training (*p* < 0.05). Heart rate was significantly reduced in both training swimming groups after a period of ischemia (*p* < 0.05). On the other hand, the anaerobic running protocol induced a significantly decreased heart rate in comparison with the aerobic running group and the sedentary group (*p* < 0.05). *Conclusions*: The data from this experimental study support many protective training effects, i.e., improved contractility, improved resting heart rate, and increased physical work capacity and exercise tolerance. Physical training in the form of anaerobic running induces greater heart preconditioning for reperfusion injury in comparison with anaerobic swimming training.

## 1. Introduction

According to the World Health Organization, the most common causes of death in many parts of the world are different cardiovascular diseases (CVDs). Reports suggest that among various cardiovascular diseases, the most frequent cause of death lies in ischemic heart disease (IHD) [1,2]. IHD is often caused due to coronary artery stenosis, leading to insufficient blood circulation and decreased blood perfusion of the heart, resulting in ischemia, reduced metabolism, and irregular cardiac function [3,4,5,6,7,8].

The treatment and therapy of IHD in recent decades represent one of the biggest tasks of the health community, where the emphasis is mainly on a pharmacological approach, i.e., the development of a large number of pharmacological agents in the control and treatment of ischemic heart disease [9]. Apart from the therapeutic problem, it is well known that the prevention of a possible ischemic event can be an important factor in reducing the consequences of this disease, both in terms of the clinical picture and in terms of treatment costs. Therefore, in order to prevent the myocardium from I/R injury, a preconditioning procedure was developed, which is a procedure for the protection and prevention of the myocardium from impending ischemic and reperfusion damage, and which is carried out using pharmacological and non-pharmacological principles [10]. One of the non-pharmacological types of preconditioning—physical activity—was shown to be potentially the most effective with numerous promising results in this field [10,11,12].

A known fact is that regular aerobic exercise can promote metabolism, regulate hormone equilibrium, improve cognitive function, and is a recommended factor in controlling cardiovascular disease. Improving systemic circulation, increasing cardiopulmonary function, increasing coronary artery perfusion, and improving cardiac stress tolerance are all effects of regular exercise [13,14]. Physical exercise can lead to an increase in ventricular dimensions and myocardial mass, and it can increase both cardiac output and stroke volume [15]. Exercise training and its effects on myocardial ischemia (MI) were first reported in a study by McElroy et al. [16]. Their study found that exercising could reduce the infarct size after MI due to it inducing the formation of new blood vessels in rat hearts [16]. For animals, the most commonly employed interventional exercising methods are running on a treadmill and swimming. Studies showed that treadmill training in rats increases antioxidant enzyme levels and improves blood-pumping capacity recovery following MI [17].

Regular physical exercise can lead to an increase in antioxidant enzyme levels in muscles [18]. Exposure to cold, whether the medium is cold air or water, is a significant environmental stressor. Since cold mediums trigger many different responses in living organisms, both thermoregulatory and chemical, cold stimulation is used as a form of physical therapy [19]. Following up on the last statement, water immersion is a popular recovery method, especially after long and hard training sessions. It is most commonly used by athletes for reducing muscle pain or accelerating fatigued muscles’ recovery, but it is not limited to them since it is also recommended for older people to modify their lifestyles [20]. Exposure to different temperatures and hydrostatic pressure are the factors responsible for triggering various physiological changes. Exercising in cold environments has provided significant evidence of increased usage of body fat as a more prominent energy source and an increased VO_2_ demand with both immediate and long-term physiological and biochemical consequences [21]. During immersion in cold water, a decrease in the skin, subcutaneous, muscle, and sometimes even rectal temperature results from increased metabolism and oxygen usage to maintain adequate temperatures of vital organs [22]. While the effects of acute and regular exercise on oxidative stress and antioxidant systems were investigated in several studies, only a few have confirmed that swimming increases oxidative stress in humans and that periodical immersion in cold water could be used to improve immune system responses and antioxidant protection [23].

Studies show that swimming training, especially before MI, can increase cardiac antioxidant capacity in rats and improve their systolic function [23,24]. Increased cardiac arteriole density and improved cardiac function during the remodeling phase were also reported as an effect of swimming preconditioning in rats [24].

Also, using the Langendorff technique allows for the induction of ischemia, arrhythmia, and hypoxia to various degrees, which makes it a unique tool for the study of pathological cardiac conditions. This model provides for testing the different procedures that change the myocardial function and provides for comparison with the intact function of the working myocardium and the coronary vessels to obtain conclusions based on the measurement of a variety of parameters [25].

This study aimed to examine the influence of different training modalities, such as swimming and running, as physiological preconditioning for the prevention of isolated rat heart ischemia/reperfusion injury. Also, this study looks to answer questions on how different modalities of physical training (aerobic and anaerobic training) and intensity (moderate and high intensity) could impact the prevention or reduction of functional heart damage after ischemia.

## 2. Materials and Methods

### 2.1. Ethical Concerns

This experimental study was approved by the Ethics Committee for the Welfare of experimental animals, Faculty of Medical Sciences, University of Kragujevac, (No. 01-10540/3). All procedures followed the Guidelines for the Care and Use of Laboratory Animals (GLP). According to these guidelines, we followed all procedures that reduced suffering for the animals during laboratory testing: reduced number of animals needed in a procedure; refined procedures to cause the least possible distress to the animals; acclimatized the animals to new environments; well-trained staff; positive reinforcements; and helpful tools (analgesics, anesthetics, and non-pharmacological strategies) [26].

### 2.2. Experimental Animals

This study was conducted on 60 male Wistar albino rats (6 weeks old, with an average body weight of 200 ± 20 g). Their habitat in the Institute of Cardiovascular Physiology consisted of strictly controlled environmental conditions such as consistent room temperature (22 ± 2 °C) with established day/night cycles lasting 12 h and an abundance of food and water to be taken freely (ad libitum). The Wistar outbred albino rat is the most popular rat model due to its multi-purpose characteristics, productive performances, and cost benefits, making it a powerful species for clinical medical research in physiology and long studies.

The animals (n = 60) used in this study were healthy and not under the effects of drugs or supplements. They were randomly divided into three experimental groups (12 animals per group):

1. CTRL group—a sedentary control group of rats that were not exposed to physical training;
2. sAeT—a group that underwent aerobic swimming conditioning using a swimming protocol for 8 weeks;
3. sAnT—a group that underwent anaerobic swimming conditioning for 8 weeks;
4. rAeT—a group that underwent aerobic running conditioning using a running protocol for 8 weeks;
5. rAnT—a group that underwent anaerobic running conditioning using a running protocol for 8 weeks.

### 2.3. Aerobic and Anaerobic Swimming Protocol

The swimming protocols were conducted in a specially constructed glass tank (80 cm × 60 cm × 100 cm) with an installed electric heater used for maintaining water temperature (37 ± 1 °C) and a pump for continuously making waves to prevent floating instead of swimming. The animals were under supervision during the entire duration of the exercises. The training protocol began every day early in the morning (9:00 a.m.) for all training sessions. Simultaneously, a maximum of six (6) rats were placed in the water tank [27,28].

In the aerobic swimming protocol (AeS), the corresponding group was swimming for five (5) days per week for nine (9) weeks continuously. The first week of the protocol was an adaptation period with the animals continuously swimming for 20 min on the first day, with the swimming time increasing by 10 min daily and ending with a 60 min swimming session on the fifth day. The exercise duration was constant from the second week onwards (60 min/day, 5 days/week), with no additional weight added, so the required level of physical exercise was just below the recommended anaerobic level [28,29].

To confirm that we reached an anaerobic level of lactates, we used the following swimming protocol (ArS). The anaerobic threshold of lactate was set at 4.5 mmol/L, and all values below 3.5 mmol/L were set as aerobic.

The protocol consisted of swimming for 60 min daily for five (5) days per week during the first two weeks as an adaptation period. The following three weeks consisted of 15 min swimming sessions where each animal was equipped with lead weights corresponding to 6.5% of their weight. During the last four weeks of the protocol, the rats underwent 60 min swimming sessions again equipped with lead weights corresponding to 6.5% of their body weight. At the end of each week of training, we measured the lactate levels in exercising rats via tail vein punction using a portable lactate monitor and a lancet. To simulate the same stress levels that our exercising groups of animals had, the rats from the control group were also put in water for 1 min per day/5 days a week until the end of the training period [27].

### 2.4. Swimming Pool for Experimental Animals

Swim-training was performed in a glass pool (60 cm × 100 cm × 75 cm), with the water depth 60 cm and the temperature of the aquatic environment 37 ± 1 °C. Training was performed by two groups simultaneously with continuous monitoring of the training and the temperature of water environment. In order to experience the same experimental conditions, rats from CTRL group were placed into a separate water tank (depth 5 cm) for five minutes at the same water temperature [28].

### 2.5. Aerobic and Anaerobic Running Protocol

After the adaptation period, rats subjected to moderate intensity running would run 60 min a day for the next five weeks, gradually increasing the running speed (10–13 m/min) so that in the last week the rats ran at a speed of 15 m/min [26,27]. The total duration of this protocol is 6 weeks including adaptation period.

High Intensity Interval Training Protocol (High Intensity Interval Running) means that after the adaptation period, rats subjected to high-intensity interval running would run at an intensity of 45 m/min for 30 s from the second week, and after a 3 min rest, this procedure would be repeated in four more cycles. During the next three weeks, both the speed and the duration of running was gradually increased so that in the last week, the rats would run at a speed of 55 m/min for 90 s in five cycles [27]. In order to evaluate and confirm the aerobic or anaerobic type of running in animals from both groups, lactates were measured after each running session (at least three times a week) using a commercial lactometer by puncturing the tail vein [27].

### 2.6. Treadmill for Small Experimental Animals

Exercise protocol was performed by Treadmill for rats (ELUNIT Medical Equipment), customized for anatomical and physiological characteristics of small experimental animals (power supply 220 V, 50 Hz; number of trails for running: 4; speed control 2–50 m/min with a resolution of 0.1 m/min; bars which deliver mild electric shock of 0–0.5 mA with a 1–2 s interpulse interval), connected with Treadmill software (animal treadmill for rats—TM S01) to monitor speed continuously. Mild electric shock was activated when rats stopped running but without causing stress to animals [29,30].

### 2.7. Ex Vivo Estimating Myocardical Function of Wistar Rats

We excised and then perfused the hearts of all the exercising animals (n = 60, 12 in each experimental group) on Langendorff apparatus (Experimetria Ltd., 1062 Budapest, Hungary) [25,31]. After a brief anesthetization period using ketamine (10 mg/kg) and xylazine (5 mg/kg), cervical dislocation was performed and the animals were sacrificed (Schedule 1 of the Animals/Scientific Procedures, Act 1986, UK). After the ensuing midline thoracotomy, the hearts were extracted and dipped in cold isotonic saline before being mounted on the Langendorff apparatus for retrograde perfusion under constant coronary perfusion pressure (CPP of 70 cmH_2_O). For the perfusion the Krebs–Henseleit buffer was used (in mmol/L: NaCl 118, KCl 4.7, CaCl_2_ × 2H_2_O 2.5, MgSO_4_ × 7H_2_O 1.7, NaHCO_3_, KH_2_PO_4_ 1.2, glucose 11, and pyruvate 2). The buffer was then warmed up to 37 °C while maintaining a pH of 7.4 and infused with 95% O_2_ and 5% CO_2_. After the successful mount, a stabilization period of circa 20 min at CPP of 70 cm H_2_O transpired, after which a 30 min global ischemia session ensued. Following the ischemia session, a 30 min reperfusion period started. Different parameters [25] for assessing myocardial function were measured with a sensor placed in the left heart ventricle:

1. Maximum rate of pressure development in LV (dp/dt max);
2. Minimum rates of pressure development in LV (dp/dt min);
3. Systolic left ventricle pressure (SLVP);
4. Diastolic left ventricle pressure (DLVP);
5. Heart rate (HR);
6. Coronary flow (CF) was measured flowmetrically.

### 2.8. Statistical Analysis

Values are expressed as the mean ± standard error of mean. The well-known Shapiro–Wilk test was the method used to test the normality of the data. For testing the differences between groups in each parameter, one-way ANOVA and Tukey post hoc test were used to confirm the differences. Normality tests and analytical tests were conducted using the statistical software SPSS version 26. The statistical significance threshold was set at 0.05.

## 3. Results

**Figure 1:**
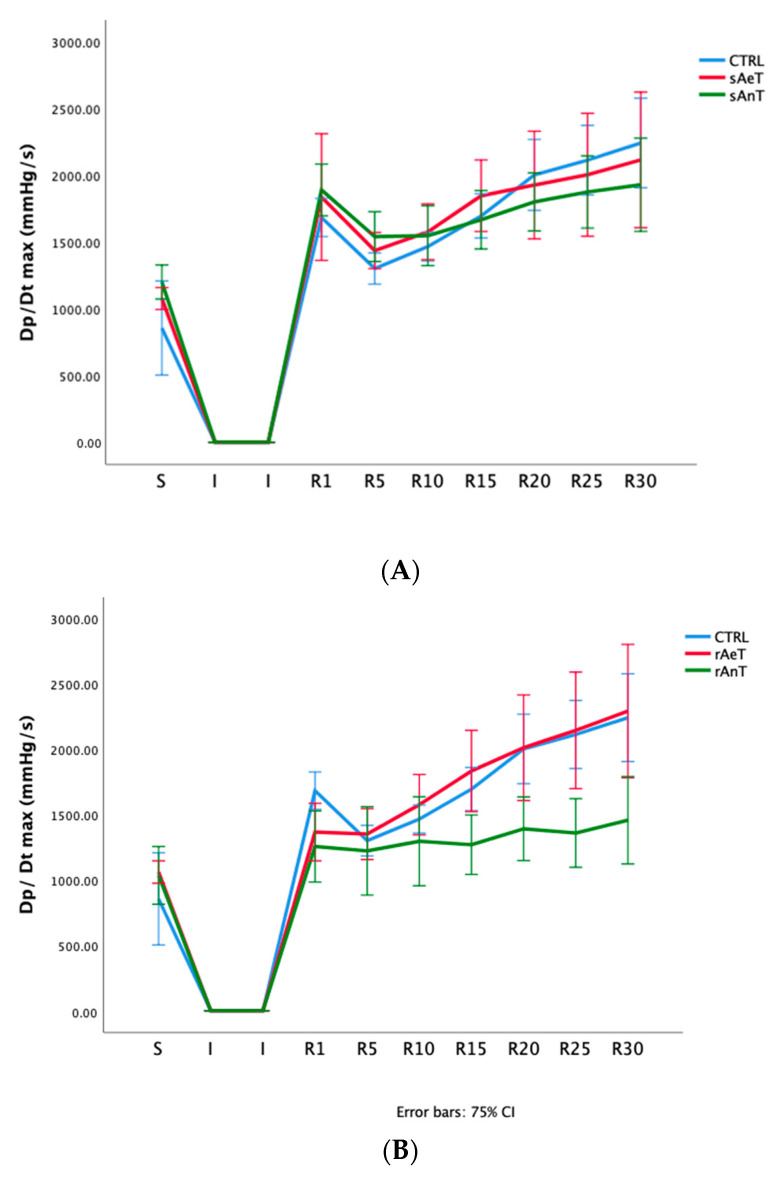
Values of maximum rate of development of left ventricular pressure in (**A**): CTRL, sAeT, and sAnT groups; and (**B**): CTRL, rAeT, and rAnT. Results are presented as mean ± standard error of mean.

**Figure 2:**
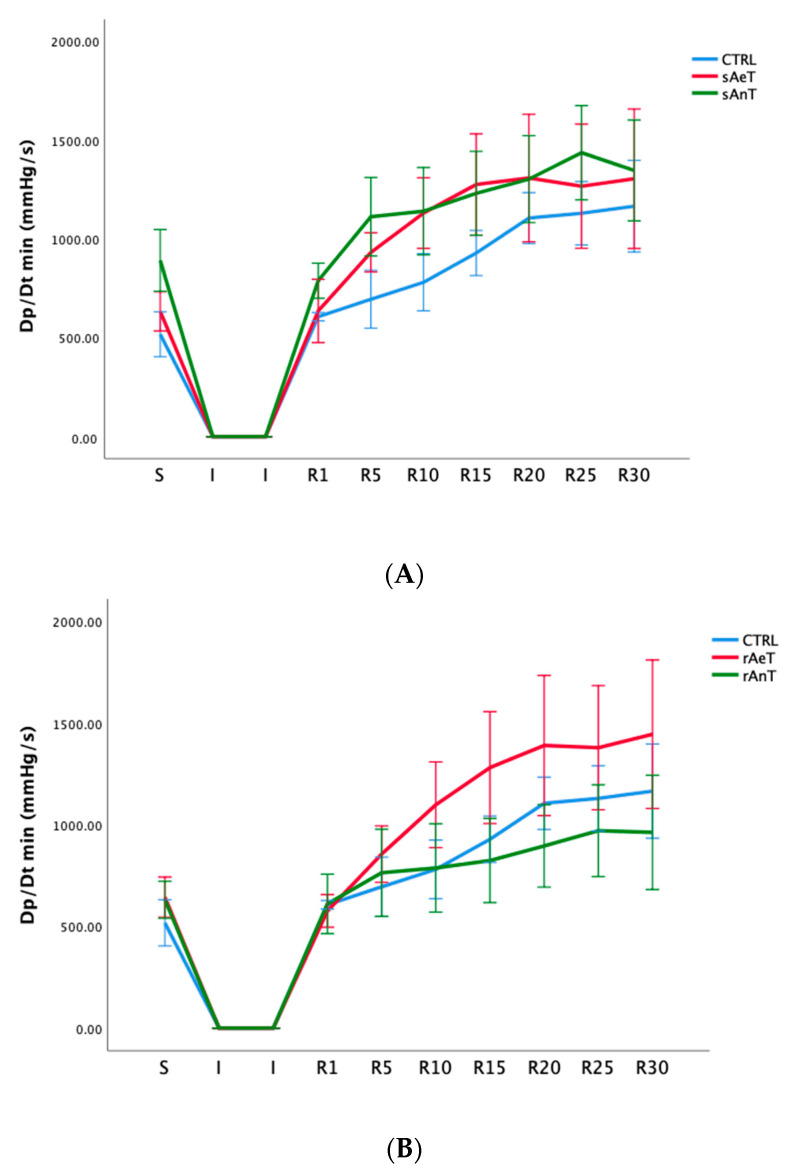
Values of minimum rate of development of left ventricular pressure (**A**): CTRL, sAeT, and sAnT groups; and (**B**): CTRL, rAeT, and rAnT. Results are presented as mean ± standard error of mean.

**Figure 3:**
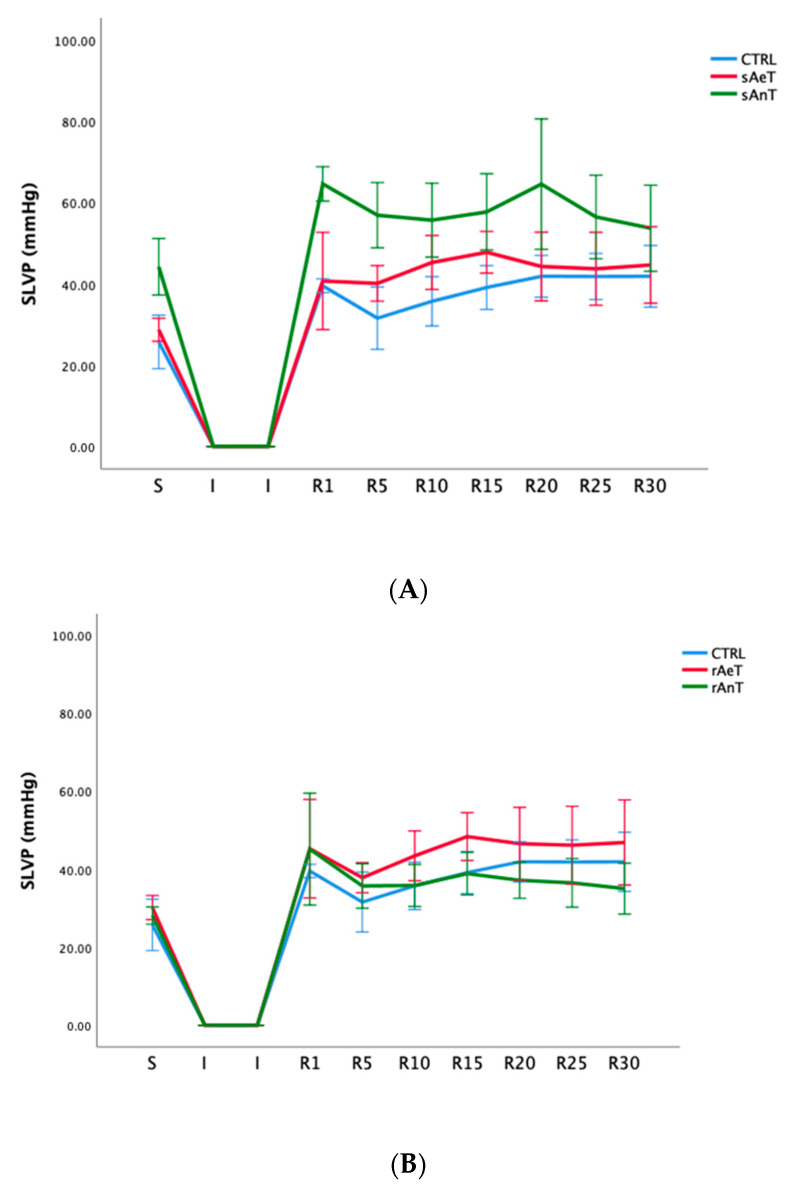
Values of systolic left ventricular pressure in (**A**): CTRL, sAeT, and sAnT groups; and (**B**): CTRL, rAeT, and rAnT. Results are presented as mean ± standard error of mean.

**Figure 4:**
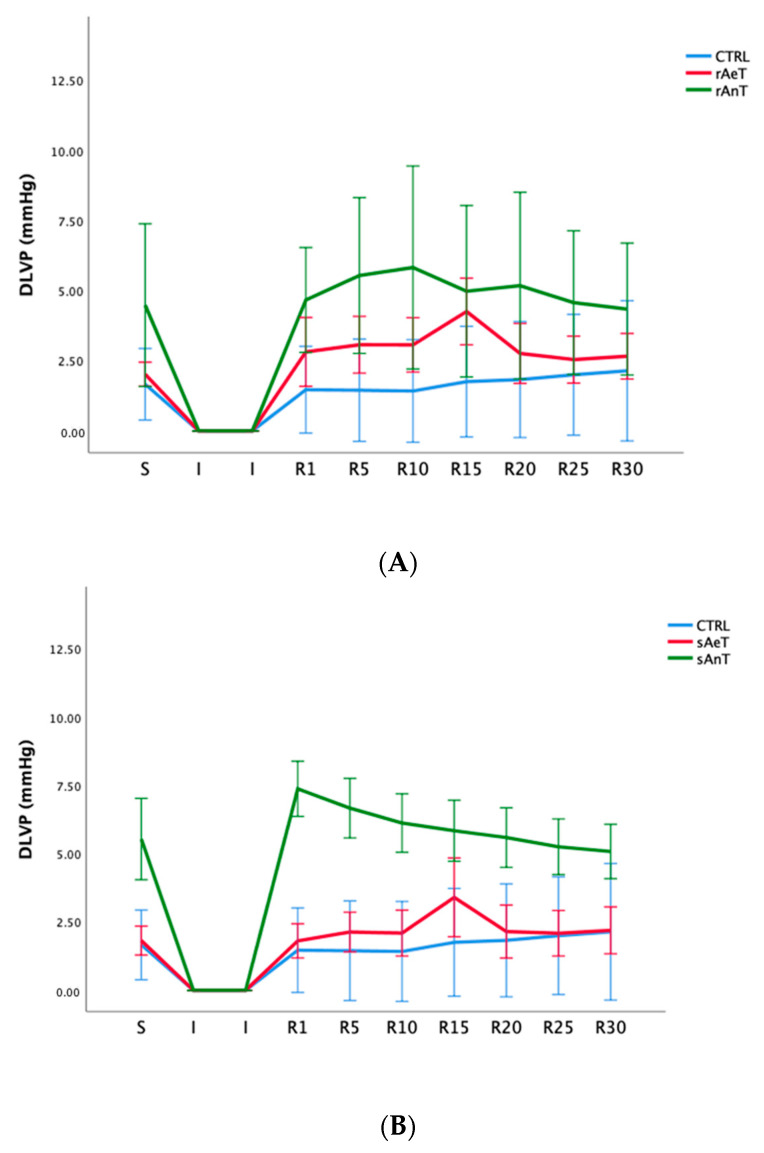
Values of diastolic left ventricular pressure in (**A**): CTRL, sAeT, and sAnT groups; and (**B**): CTRL, rAeT, and rAnT. Results are presented as mean ± standard error of mean.

**Figure 5:**
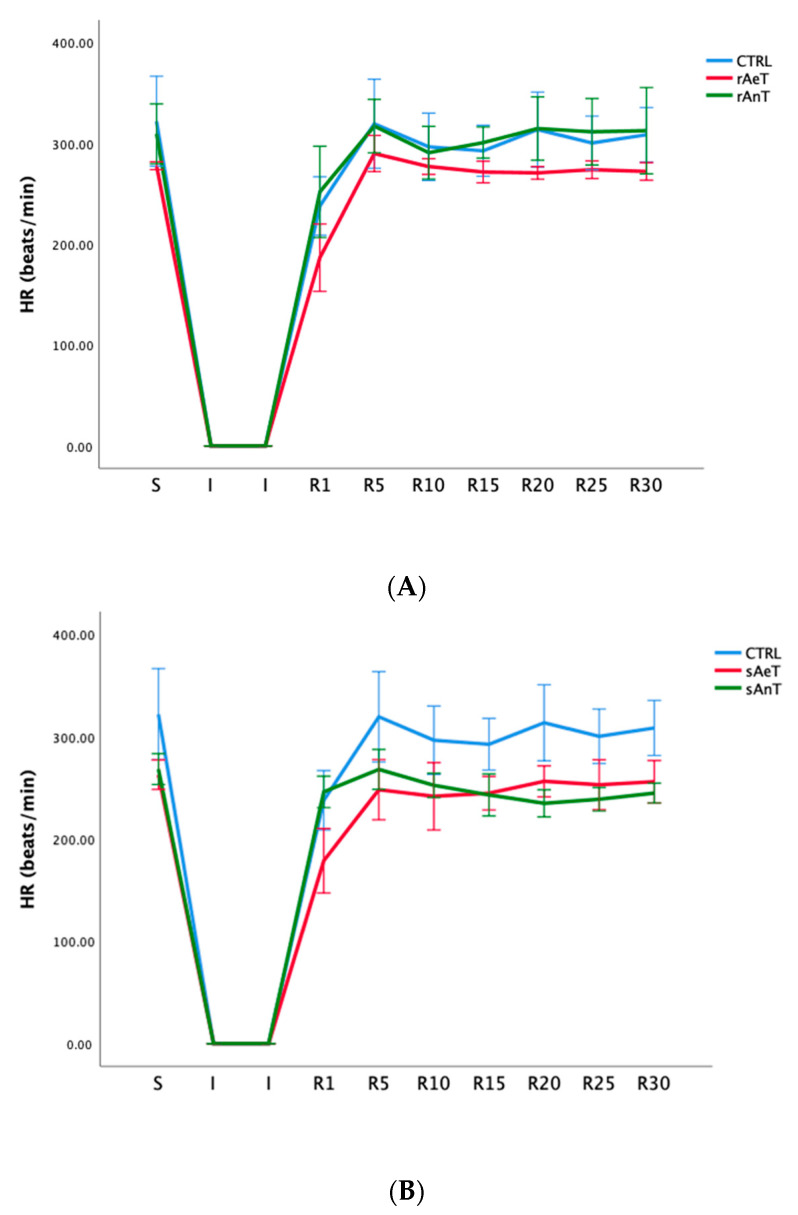
Values of heart rate (HR) in (**A**): CTRL, sAeT, and sAnT groups; and (**B**): CTRL, rAeT, and rAnT. Results are presented as mean ± standard error of mean.

**Figure 6:**
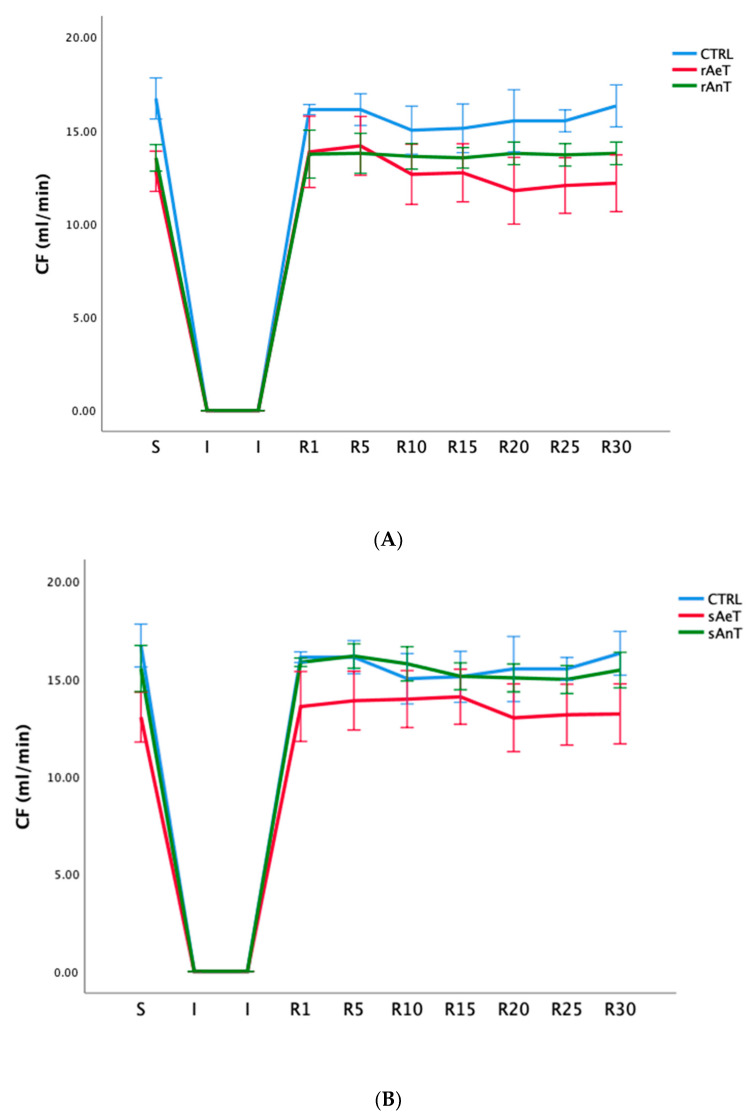
Values of coronary flow (CF) in (**A**): CTRL, sAeT, and sAnT groups; and (**B**): CTRL, rAeT, and rAnT. Results are presented as mean ± standard error of mean.

In Figure 1, Figure 2, Figure 3, Figure 4, Figure 5 and Figure 6 the dynamics of the parameters of myocardial function measured in ex vivo conditions are shown. The main parameters of heart contractility, dp/dt max, and dp/dt min are significantly changed in comparison with the CTRL values. At the beginning of the reperfusion period (R1–R15), the dp/dt max was increased in the sAnT group, and later increased in the sAeT group in comparison with the CTRL (Figure 1A). On the other hand, the dp/dt max with the running protocol was significantly decreased in the rAnT group in comparison with the aerobic running and sedentary groups of animals (Figure 1B). From Table 1 we can observe that in most of the measured points, the changes to the dp/dt max in both the swimming and running protocols were altered in comparison with the control conditions (Table 1).

Further, the second parameter of heart contractility, named dp/dt min, was also significantly altered during different types and intensities of training. That means that during swimming, the dp/dt min in the R period was similarly increased in both groups (sAeT and sAnT) and during running, the aerobic protocol induced an increased contractility (Figure 2 and Table 2).

Systolic left ventricular pressure was significantly increased during the anaerobic protocol with swimming and during the anaerobic protocol with running. Both protocols, aerobic and anaerobic, induced an elevation in SLVP in both types of training (Figure 3 and Table 3).

DLVP was similarly significantly increased in the anaerobic protocols of swimming and running, and also increased contractility and left ventricular pressure during training in comparison to the sedentary (control) conditions (Figure 4 and Table 4).

Very interestingly, heart rate was significantly reduced in both swimming training groups after a period of ischemia. On the other hand, the anaerobic running protocol induced a significantly decreased heart rate in comparison with the aerobic running and sedentary groups (Figure 5, Table 5).

Finally, for the parameter of coronary circulation/coronary flow, we measured flowmetrically and manually for each point. We observed that the anaerobic swimming training induced a decreased heart rate in the R period in comparison with the aerobic and control groups. On the contrary, running both the aerobic and anaerobic training induced a significantly decreased heart rate during the R period in comparison with the CTRL group (Figure 6, Table 6).

## 4. Discussion

This experimentally designed study, using in vivo and ex vivo conditions, was aimed at examining the influence of different training modalities, such as swimming and running, as physiological preconditioning methods on rat heart ischemia/reperfusion injury. In addition, this preclinical research provided an answer to questions about how different intensities and types of physical training (aerobic/anaerobic or swimming/running) could improve function or reduce heart dysfunction after myocardial ischemia.

Our preclinical research was conducted on 60 male adult non-obese Wistar albino rats which are confirmed to be good and reliable models for testing the exercise protocols on the cardiovascular system.

Animal models of acute and chronic exercise are commonly used in research. Often the acute exercise test is used in various combinations, such as genetic, pharmacological, and other manipulations. All the combined protocols could provide a significant result in the cardiovascular response to exercise and detect changes in cardiovascular function that may not be evident at rest [25,31]. On the other hand, chronic exercise conditioning models are used to study the cardiac phenotypic response to regular exercise protocols and may provide information on novel pathways mediating cardiovascular health [31]. Although most of the benefits of exercise on the cardiovascular system are known, the exact mechanism by which aerobic and anaerobic training could improve myocardial function is still unclear. In this sense, our training models, both swimming and running in a chronic way, represent the gold standard in evaluating the effects of exercises on the cardiovascular system.

### 4.1. Swimming vs. Running: The Effects on Heart Function and Cardiovascular Health

This unsurpassed question about the comparison of swimming and running in terms of benefits is actually still current. It is most important to find the right type of physical activity that will achieve the best fitness goals and promote health. Currently, the most popular aerobic physical activities are running and swimming. Whatever the personal goals, both of them promote conditioning, losing weight, improving cardiovascular health, burning calories, gaining muscle, and improving bone density [32]. In addition, swimming could burn more calories in comparison with running the same distance. Swimming is a complete body workout, which results in burning more calories compared to other activities. Also, the risk of injury is different, and swimming is the type of training that people with problems with joint function can undertake because it has a low impact on joints. With running, most injuries can be prevented, but most of the problems come from frequent pain and soft tissue injuries.

On a molecular level, aerobic metabolism generates more adenosine three phosphate (ATP) and relies more on oxygen, while anaerobic metabolism does not need oxygen but creates two ATP molecules per one molecule of glucose. In addition, both types of activity need to produce cellular energy [32].

In our study, we confirmed the differences between the effects of the type of activity on changing the myocardial contractility after a long period (8 weeks) of preconditioning. For estimating the main course of cardiac contractility, the most important are the parameters of maximum and minimum rate of development of left ventricular pressure and systolic pressure (dp/dt max, dp/dt min, and SLVP).

In Figure 1, Figure 2, Figure 3, Figure 4, Figure 5 and Figure 6 the dynamics of the parameters of myocardial function measured in ex vivo conditions are shown. The main parameters of heart contractility, dp/dt max, and dp/dt min are significantly changed in comparison with the CTRL values. In the beginning of the reperfusion period (R1–R15), the dp/dt max was increased in the sAnT group, but also later increased in the sAeT group in comparison with the CTRL (Figure 1A). On the other hand, the dp/dt max when using the running protocol was significantly decreased in the rAnT group in comparison with the aerobic running and sedentary groups of animals (Figure 1B). From Table 1 we can observe that in most of the measured points, the changes to the dp/dt max with the swimming and running protocols were altered in comparison with the control conditions (Table 1). Further, the second parameter of heart contractility, named as dp/dt min, was also significantly altered during the different types and intensities of training. That means that during swimming, the dp/dt min in the R period was similarly increased in both groups (sAeT and sAnT) and during running, the aerobic protocol induced more increased contractility (Figure 2 and Table 2). Systolic left ventricular pressure was significantly increased during the anaerobic protocol for swimming, and during the anaerobic protocol for running. Both protocols, aerobic and anaerobic, induced an elevation of SLVP in both types of training (Figure 3 and Table 3). Definitely, the aerobic modality of training when both swimming and running have shown the ability of improving cardiac function and reducing reperfusion injury after global ischemia in an animal model. The differences in the effects of anaerobic and aerobic activity in our results can be explained by the well-known fact that in cells, aerobic respiration occurs within the mitochondria and anaerobic respiration occurs in the cytoplasm of a cell [33,34]. This evidence is also in accordance with previous studies, which confirmed the heart hypertrophy induced by physical activity. After a century of experimental and clinical investigations, exercise-induced cardiac hypertrophy is a result of training improved contractility and that is an expected adaptation of regular physical training [35,36,37].

Baptista et al. evaluated the effects of treadmill running and swimming in rats [38]. Their study was performed on the normal Wister rat to evaluate the effects of two distinct moderate/long-term aerobic training protocols, treadmill running and the swimming, on several important parameters related to cardiovascular (CV) physiological adaptations, namely: lipid profile, hemorheological measures, lipid peroxidation, peripheral serotonergic system (SS) modulation, and sympathetic nervous system (SNS) activation. In both groups of training, an HDL-c increment versus the sedentary control was demonstrated. There was a noticeable increase in ADP-induced platelet aggregation in the exercised rats, together with higher PDW and MPV values. They concluded that treadmill running was more influential than swimming when concerned with peripheral SS modulation, while swimming was more important on SNS activation, thus recommending a judicious choice of the protocol to be tested in works which make use of rat models of exercise to study physiological or pathophysiological conditions [39]. Similar to these conclusions, our results confirmed that running induced more intensive improvement in cardiac contractility in comparison with the swimming.

The mechanism for inducing these adaptations remains unknown, but it could be due to the hypoxic environment when swimming [37,39]. In contrast, endurance running programs involving rodents (as well as other species) suggest relatively little change in the biochemical and functional properties of the contractile system [39]. In addition to that, a great advantage of this modality is that the exercise training requires only minimal investigator intervention and does not require aversive stimuli (e.g., electric shocks or air jets) to motivate the animals to run in the wheels.

### 4.2. Aerobic vs. Anaerobic Exercise: The Effects on Heart Function and Cardiovascular Health

Both aerobic and anaerobic exercise are important for optimal health. As previously mentioned, aerobic metabolism, known as “cardio”, generates more adenosine three phosphate (ATP) and relies more on oxygen, while anaerobic metabolism does not need oxygen but creates two ATP molecules per one molecule of glucose [32]. The optimization of the physiological mechanisms of exercise and their subsequent impact on cardiovascular health remains a subject of ongoing uncertainty, both in animal models and clinical practice.. Good models of exercise testing are animal studies. In most cases, animal-based cardiovascular exercise modalities include treadmill running, swimming, and voluntary wheel running with a series of intensities, times, and durations [38,39,40,41]. Those using animals include small rodents (e.g., mice and rats) and large animals (e.g., rabbits, dogs, goats, sheep, pigs, and horses). Depending on the research goal, each experimental protocol should also describe whether its respective exercise treatment can produce the anticipated acute or chronic cardiovascular adaptive response [38,40].

Kent et al. [42] presented multiple lines of cardiovascular discovery using exercise models that were initiated in animals as the fundamental step for designing and testing explicit hypotheses in humans. One of those types is our study.

In our study, we evaluated the differences in the aerobic and anaerobic physical training in both modalities, swimming and running, in relation to the preconditioning of heart function. We confirmed that the anaerobic running protocol strongly induces an increased contractility in rat hearts (increasing the dp/dt max and dp/dt min), while the aerobic swimming protocol was more powerful in comparison with the aerobic running (Figure 1, Figure 2, Figure 3, Figure 4, Figure 5 and Figure 6). Our report is a good way to present an overall picture of scientific research studies using animal models with a focus on the different training modalities that we examined. One thing is certain, the tested groups exhibit varying exercise capacities and maximum oxygen uptake, leading to divergent results in cardiac contractility.. Geenen and colleagues compared aerobic training to anaerobic training and found that multiple training-induced cardiovascular and hormonal adaptations produced in the rat differed significantly [39,42]. Anaerobic training (HIIT) increased maximal exercise performance and reduced the blood lactate responses more compared with free-wheel or aerobic running. These differential cardiovascular, skeletal muscle, and metabolic training responses across the three exercise modalities should be considered carefully, especially in comparison to the aerobic protocols of swimming and running.

Very interestingly, heart rate was significantly reduced in both swimming training groups after the ischemia period. On the other hand, the anaerobic running protocol caused a significant decrease in heart rate compared to the aerobic running and sedentary groups (Figure 5, Table 5). Finally, with the coronary circulation parameter—coronary flow—we measured the flow metrically and for each point manually. We observed that the anaerobic swimming training caused a decrease in heart rate in the R period compared to the aerobic and control groups. On the contrary, running both aerobically and anaerobically caused a significant decrease in heart rate during the R period compared to the CTRL group (Figure 6, Table 6).

These interesting results (that anaerobic running training decreased heart rate and anaerobic swimming training reduced coronary flow) occur due to all the previously mentioned differences in the physiological heart response to aerobic/anaerobic physical training [37,38,39,40,41,43].

## 5. Conclusions

All data from this experimental study support many training effects: improved contractility, resting heart rate, and increased physical work capacity and exercise tolerance. Anaerobic running physical training induces greater heart preconditioning to reperfusion injury in comparison with anaerobic swimming training. The physiological response with aerobic swimming physical training is more valuable for improving exercise tolerance and coronary circulation. Future directions for testing the preconditions strategies will be focused on testing the effects of adjuvant antioxidant agents in combination with different physical training protocols.

## Figures and Tables

**Table 1:** Comparison of changing dp/dt max in all experimental groups with CTRL *.

Group	sAeT	sAnT	rAeT	rAnT	*p*-Value
R1	35.80	44.93	39.20	−22.57	*p* < 0.05 ^a–f^
R5	6.63	14.35	2.23	−17.02	*p* < 0.05 ^a–f^
R10	14.17	26.53	58.28	3.65	*p* < 0.05 ^a–f^
R15	30.48	36.90	83.96	15.03	*p* < 0.05 ^a–f^
R20	11.45	41.99	113.00	28.43	*p* < 0.05 ^a–f^
R25	18.32	48.58	131.00	4.58	*p* < 0.05 ^a–f^
R30	21.92	58.36	161.28	9.15	*p* < 0.05 ^a–f^

* The values are obtained from ratio of stabilization (S) and one of the following reperfusion points (R1–R30), by using calculation 100 − ([value] Rx − [value] S) × 100 = [value]%. Statistical analysis was performed using ANOVA and Tukey post hoc test as follows: a = sAet vs. sAnT; b = sAeT vs. rAeT; c = aAet vs. rAnT; d = sAnT vs. rAeT; e = sAnT vs. rAnT; f = rAeT vs. rAnT.

**Table 2:** Comparison of changing dp/dt min in all experimental groups with CTRL *.

Group	sAeT	sAnT	rAeT	rAnT	*p*-Value
R1	−23.14	−39.30	−5.03	−45.67	*p* < 0.05 ^a–f^
R5	1.34	−7.72	−11.16	−27.95	*p* < 0.05 ^a–f^
R10	11.41	16.83	62.03	−10.82	*p* < 0.05 ^a–f^
R15	20.89	29.56	88.47	−16.69	*p* < 0.05 ^a–f^
R20	−1.75	31.86	127.31	−2.09	*p* < 0.05 ^a–f^
R25	−3.26	32.35	115.90	−8.57	*p* < 0.05 ^a–f^
R30	−4.07	28.41	147.36	−34.50	*p* < 0.05 ^a–f^

* The values are obtained from ratio of stabilization (S) and one of the following reperfusion points (R1–R30), by using calculation 100 − ([value] Rx − [value] S) × 100 = [value]%. Statistical analysis was performed using ANOVA and Tukey post hoc test as follows: a = sAet vs. sAnT; b = sAeT vs. rAeT; c = aAet vs. rAnT; d = sAnT vs. rAeT; e = sAnT vs. rAnT; f = rAeT vs. rAnT.

**Table 3:** Comparison of changing SLVP in all experimental groups with CTRL *.

Group	sAeT	sAnT	rAeT	rAnT	*p*-Value
R1	12.23	18.26	107.15	−19.61	*p* < 0.05 ^a–f^
R5	3.66	2.68	4.87	−1.66	*p* > 0.05
R10	4.75	19.73	43.67	12.52	*p* > 0.05
R15	39.11	22.75	62.49	−0.54	*p* < 0.05 ^a–f^
R20	−14.81	24.50	72.93	9.91	*p* < 0.05 ^a–f^
R25	−18.12	24.05	72.93	−11.90	*p* < 0.05 ^a–f^
R30	−19.11	19.38	80.38	−20.30	*p* < 0.05 ^a–e^

* The values are obtained from ratio of stabilization (S) and one of the following reperfusion points (R1–R30), by using calculation 100 − ([value] Rx − [value] S) × 100 = [value]%. Statistical analysis was performed using ANOVA and Tukey post hoc test as follows: a = sAet vs. sAnT; b = sAeT vs. rAeT; c = aAet vs. rAnT; d = sAnT vs. rAeT; e = sAnT vs. rAnT; f = rAeT vs. rAnT.

**Table 4:** Comparison of changing DLVP in all experimental groups with CTRL *.

Group	aAeT	sAnT	rAeT	rAnT	*p*-Value
R1	−59.31	8.14	160.87	−23.88	*p* < 0.05 ^a–f^
R5	−46.90	5.16	137.50	6.34	*p* < 0.05 ^a–d^
R10	381.38	−4.47	141.85	28.90	*p* < 0.05 ^a–f^
R15	−62.76	−6.77	154.35	9.55	*p* < 0.05 ^a–f^
R20	−66.90	−11.35	139.67	16.91	*p* < 0.05 ^a–f^
R25	−48.28	−13.88	100.54	−5.64	*p* < 0.05 ^a–f^
R30	−47.59	−18.92	113.04	−12.61	*p* < 0.05 ^a–f^

* The values are obtained from ratio of stabilization (S) and one of the following reperfusion points (R1–R30), by using calculation 100 − ([value] Rx − [value] S) × 100 = [value]%. Statistical analysis was performed using ANOVA and Tukey post hoc test as follows: a = sAet vs. sAnT; b = sAeT vs. rAeT; c = aAet vs. rAnT; d = sAnT vs. rAeT; e = sAnT vs. rAnT; f = rAeT vs. rAnT.

**Table 5:** Comparison of changing HR in all experimental groups with CTRL *.

Group	aAeT	sAnT	rAeT	rAnT	*p*-Value
R1	−9.13	−12.47	−23.55	−52.97	*p* < 0.05 ^d,e,f^
R5	−5.03	−6.32	16.24	−7.75	*p* < 0.05 ^e^
R10	0.47	−15.07	4.51	−28.41	*p* < 0.05 ^a–f^
R15	−8.74	−18.20	0.20	−17.09	*p* < 0.05 ^a–f^
R20	−0.46	−20.83	1.12	−1.22	*p* < 0.05 ^a^
R25	0.47	−18.53	4.01	1.88	*p* < 0.05 ^a,b,e^
R30	1.67	−12.47	2.31	7.84	*p* < 0.05 ^a,b,e^

* The values are obtained from ratio of stabilization (S) and one of the following reperfusion points (R1–R30), by using calculation 100 − ([value] Rx − [value] S) × 100 = [value]%. Statistical analysis was performed using ANOVA and Tukey post hoc test as follows: a = sAet vs. sAnT; b = sAeT vs. rAeT; c = aAet vs. rAnT; d = sAnT vs. rAeT; e = sAnT vs. rAnT; f = rAeT vs. rAnT.

**Table 6:** Comparison of changing coronary flow (CF) in all experimental groups with CTRL *.

Group	aAeT	sAnT	rAeT	rAnT	*p*-Value
R1	−8.33	−14.89	14.29	−12.07	*p* < 0.05 ^b,e^
R5	10.00	−4.26	11.43	−6.90	*p* < 0.05 ^b,e^
R10	16.67	−4.26	−30.00	0.00	*p* < 0.05 ^b,e^
R15	20.00	−12.77	−28.57	3.45	*p* < 0.05 ^b,e^
R20	−6.67	−10.64	−35.71	3.45	*p* < 0.05 ^b,d^
R25	−8.33	−10.64	−28.54	3.41	*p* < 0.05 ^b,d^
R30	−6.67	−4.26	−28.57	3.39	*p* < 0.05 ^b,d^

* The values are obtained from ratio of stabilization (S) and one of the following reperfusion points (R1–R30), by using calculation 100 − ([value] Rx − [value] S) × 100 = [value]%. Statistical analysis was performed using ANOVA and Tukey post hoc test as follows: b = sAeT vs. rAeT; d = sAnT vs. rAeT; e = sAnT vs. rAnT.

## Data Availability

All data collected during the study are available on written request immediately after publication, with no end date by editorial staff, medical staff, or participants who participated in research.
